# Influence of eye-related behavior on myopia among junior middle school students under the background of double reduction during the COVID-19 pandemic

**DOI:** 10.1186/s12889-024-18958-0

**Published:** 2024-06-07

**Authors:** Fengqin Li, Yin Li, Zhonghui Liu, Chang Xu, Huiwen Li, Ying Sun, Xin Zhang, Lei Gao

**Affiliations:** 1 Tianjin Heping Center for Disease Control and Prevention, Tianjin, China; 2 Dept. Maternal, Child and Adolescent Health, School of Public Health, Tianjin Medical University, Tianjin, China; 3 Tianjin Center for Disease Control and Prevention, Tianjin, China

**Keywords:** Eye-related behavior, Myopia, COVID-19, ‘double reduction’ policy, Junior middle school students, Latent profile analysis (LPA)

## Abstract

**Background:**

To investigate the changes in the unhealthy eye-related behaviors of junior middle school students during the COVID-19 pandemic and the double reduction policy and its relationship with myopia.

**Methods:**

Data were obtained from the 2019–2022 Tianjin Children and Youth Myopia, Common Diseases and Health Influencing Factors Survey. Latent profile analysis (LPA) and a generalized linear model (GLM) were applied to analyze the effect of eye-related behavior classes on myopia.

**Results:**

A total of 2508 junior middle school students were included. The types of eye-related behavior were categorized into the medium-healthy behavior group, heavy academic burden and near-eye behavior group, insufficient lighting group and high-healthy behavior group. Students with heavy academic burdens and near-eye behavior were more likely to develop myopia than were those in the high-healthy group (OR = 1.466, 95% CI = 1.203–1.787; *P* < 0.001).

**Conclusions:**

The dual reduction policy has a positive effect on improving unhealthy eye-related behaviors, and the prevention and control of myopia through the use of different combinations of eye-related behaviors are heterogeneous among junior middle school students. In the post-COVID-19 period, we should continue to implement a double reduction policy and formulate targeted eye-related behavior strategies to provide an important reference for the prevention and control of myopia among children and adolescents during public health emergencies in the future.

**Supplementary Information:**

The online version contains supplementary material available at 10.1186/s12889-024-18958-0.

## Background

In December 2019, coronavirus disease 2019 (COVID-19) began spreading globally, and this public health emergency poses a global threat [1]. The Chinese government began a nationwide moratorium on offline teaching at the end of January 2020 as an emergency measure to prevent the spread of the COVID-19 [2]. Over 180 million primary and middle school students live at home and study online courses via the Internet [3]. Children and adolescents living at home as a result of mid-to-long-term social isolation experience changes in behaviors such as less outdoor activity, increased screening, irregular sleep patterns and unhealthy dietary structures compared to school-based learning [4]. These COVID-19 prevention and control measures have resulted in billions of students experiencing lifestyle changes simultaneously, with dramatic changes in the eye environment and eye-related behavior compared to those experienced during the period before COVID-19. Higher screening, close work and limited outdoor activity have all been found to be associated with the development and progression of myopia [5]. Therefore, the changes in eye-related behavior in children and adolescents associated with COVID-19 may influence the development or worsening of myopia in school-aged children or both and may worsen during the COVID-19 pandemic and post-COVID-19 period, posing a great challenge for myopia prevention and control [6].

In July 2021, during the COVID-19 epidemic, the Chinese government proposed the “Opinions on Further Reducing the Burden of Homework and Off-Campus Training for Students in Compulsory Education”, i.e., the “Double Reduction” policy. The policy aims to further prevent and control myopia among children and adolescents by reducing the excessive academic burden, increasing outdoor activities, and promoting visual health among primary and middle school students [7]. After the implementation of the double reduction policy, primary and middle school students’ academic burdens significantly decreased, their physical activity and sleep hours increased, and their physical fitness improved [8]. Given this combined background, students’ unhealthy eye-related behavior may exhibit complex changes, thus affecting the development and progression of myopia. However, this raises a new question: how did the eye-related behavior of children and adolescents against the background of the double reduction policy during the COVID-19 epidemic differ from their eye-related behavior before the double reduction policy?

However, the growth and development of children and adolescents are also closely linked to refractive development, and the prevalence of myopia in junior middle school has increased significantly compared to that in primary school, making it the best time to study the impact of unhealthy eye-related behavior on the development of myopia and to evaluate strategies for its prevention [9]. Moreover, junior middle school is a critical period for physical and psychological development, as well as for the establishment of healthy behaviors [10]. Middle school students exhibit significant changes in almost all dimensions of their self-concept (academic, social, family and physical) [11], which leads to dramatic changes in individual psychology and behavior that may influence changes in eye-related behavior and hence have an impact on myopia. Therefore, in the dual context of the COVID-19 epidemic and the double reduction policy, an exploration of the changes in eye-related behavior of junior middle school students and their association with myopia could be an important guide for myopia prevention and control as well as precise interventions post-COVID-19. However, to our knowledge, there are no studies on the relationship between changes in eye-related behavior and myopia among junior middle school students with this dual background.

In addition, previous studies on eye-related behavior and myopia development have mostly adopted a variable-centered method, focusing on the evaluation of eye-related behavior in children and adolescents of different ages or genders [5]. However, the comprehensive eye-related behavior of individuals among children and adolescents is complex and diverse, and the variable-centered method might ignore the heterogeneity among groups of children and adolescents, making it difficult to understand the patterns and characteristics of their eye-related behavior. In the context of COVID-19 and the double reduction policy, the behavioral changes in different individuals are even more complex and diverse [12]. Therefore, an individual-centered latent profile analysis (LPA) can be used to classify children and adolescents with similar eye-related behaviors into the same category and to explore the relationship between latent eye-related behavioral categories and myopia [13]. Using the latent class model, it was possible not only to maintain the independence of each eye-related behavior for myopia but also to fit the combination of eye-related behaviors among children and adolescents and to observe whether there was group heterogeneity in the effect of different combinations of eye-related behaviors on myopia in different contexts among junior middle school students of different genders.

Above all, this study used LPA school health monitoring data from the same monitoring sites before and after the COVID-19 epidemic to summarize the types of students’ eye-related behavior during the four years before and after the COVID-19 epidemic to further analyze how the types of eye-related behavior were influenced by COVID-19 and by the double reduction policy and its impact on visual acuity development. We hypothesized that the COVID-19 epidemic and the double reduction policy had an impact on the potential categories of unhealthy eye-related behavior, that different subtypes of unhealthy eye-related behavior would have different effects on the prevention and control of myopia, and that the distribution of each subtype would be different among students of different genders over the four years. This study provides a scientific basis for the formulation of interventions for the management of middle school students’ visual health in the post-COVID-19 period and important reference values for the prevention and control of myopia among children and adolescents in future public health emergencies.

## Methods

### Participants

Our data were obtained from the 2019–2022 Tianjin Children and Youth Myopia, Common Diseases and Health Influencing Factors Survey. Our survey sample was drawn from junior middle school students aged 12–15 years within the Heping District of Tianjin using a stratified random cluster sampling method; i.e., the study site schools were first identified and then stratified by grade, and a random cluster sampling of teaching classes was used to constitute the study sample. Students who had completed the medical examination and questionnaire were eligible, while those who had not completed the questionnaire were excluded. A total of 2,508 junior middle school students were included in the analysis. All the students who participated in the survey signed an informed consent form.

The study was conducted under the Code of Ethics of the World Medical Association (Declaration of Helsinki). Also, the study complied with all relevant national regulations and institutional policies and had been approved by the Ethics Committee of Tianjin Center for Disease Control and Prevention.

### Questionnaire

Our survey used a student questionnaire developed by the Tianjin Health Committee and the Tianjin Centre for Disease Control and Prevention and was administered simultaneously to the medical examination sample. The questionnaire included basic information, sociodemographic factors (e.g., sex, age and family location), eye-related behaviors, health status and influencing factors about the students. We extracted entries from the questionnaire that were relevant to the study variables and scored them positively according to the design of the different entries. (see the supplementary materials)

### Investigation

The survey consisted of two parts: a visual acuity measurement and a questionnaire. The myopia screening mainly consisted of a distance visual acuity test (5 m standard logarithmic visual acuity meter) and a refraction test, the standard of which was the “Guidelines for the Prevention and Control of Myopia in Children and Adolescents” [14]. Visual acuity measurements and fully automated computerized optometry were measured by professionals at the Tianjin Eye Hospital. The standard logarithmic visual acuity scale has an illumination of 300–500 lx. Visual acuity data are based on the visual acuity of the right eye and uncorrected visual acuity less than 5.0 are myopia. Refractive examinations were performed using a standardized tabletop with fully automated computerized optometry. All students underwent non-cycloplegic autorefraction using an auto-kerato-refractor (Canon Autorefractor RK-F1, Tokyo, Japan). Three consecutive measurements were taken in both eyes by qualified Ophthalmologists with the assistants. Spherical refractive errors is measured as the degree of sphericity, delineating the nature and degree of refraction, i.e., -0.50 to + 0.50 D for emmetropia and ≤-0.50 D for myopia. Based on the results of the visual acuity measurements and fully automated computerized optometry, a professional ophthalmologist will diagnose on the spot whether the student is suffering from myopia, thus completing the myopia screening. Informed consent was obtained from the students and their parents with the assistance of the class teacher. All the investigators received uniform training, took the class as a unit, and answered questions anonymously in a collective and unified way. All of the questionnaires were completed by participants on their own, and they were all collected on site.

### Statistical analysis

In this study, each item of students’ eye-related behavior types during 2019–2022 was parameterized by LPA, and the LCM was constructed; this statistical analysis addresses the relationships between types of latent variables. The following criteria were used to determine the optimal model: Akaike information criterion (AIC), Bayesian information criterion (BIC), sample-size adjusted Bayesian information criterion (aBIC), bootstrap likelihood ratio test (BLRT), and adjusted LoMendell–Rubin likelihood ratio test (aLMR). Normal distributed variables were presented as mean ± standard deviation and binary variables were presented as frequency (percentage). Chi-square test and One-way ANOVA were used to explore the associations between those risk factors and profiles for quantitative and qualitive variables, respectively. After identifying the latent classes, generalized linear modeling (GLM) was used to analyze the vision and eye-related behavior types of the different latent class groups. Apart from LPA performed using MPlus 8.3, all analyses were conducted using SPSS 26.0, with a significance level set at α = 0.05.

## Results

### Demographics

A total of 2603 junior middle school students were recruited for this study, among whom 95 were excluded (3.65%) due to abnormal detection data, incomplete questionnaires, and other reasons. Table 1 contains basic demographic characteristics. Among the participants, 2508 were included in the final analysis; 1339 (53.39%) were male, and 1169 (46.61%) were female. The sample population was 12–15 years old, with an average age of 13.60 ± 0.89 years. There were 1670 (66.59) myopia students in the sample population.

**Table 1:** Distribution of basic demographic characteristics of participants

Variables	Number	mean ± SD/*N* (%)
Gender		
Male	1339	53.39%
Female	1169	46.61%
Age (year)		13.60 ± 0.89
Year		
2019	531	21.17%
2020	594	23.69%
2021	688	27.43%
2022	695	27.71%
Vision		
Normal vision	838	33.41%
Myopia	1670	66.59%

Note: SD refers to standard deviation

### Latent profile analysis of eye-related behavior

Table 2 shows that the information criterion indices AIC, BIC, and aBIC decreased with the increase in the number of latent classes and reached the maximum value in Model 5. According to the likelihood ratio test statistics, the entropy value reached a maximum in Model 2 (0.992), indicating that the model was the most accurate for sample classification when there were 2 latent classes. However, based on the model fitting evaluation results, sample size and conditional probability distribution of the latent class, the latent class of eye-related behavior types among children and adolescents was ultimately divided into 4 classes: class 1, class 2, class 3 and class 4.

**Table 2:** Results of latent profile model (LPM) fitting information

Model	AIC	BIC	aBIC	Entropy	*N*_1_	*N*_2_	*N*_3_	*N*_4_	*N*_5_	BLRT *p* value	aLMR *p* value
1	71314.931	71454.785	71378.530		2508						
2	68766.683	68982.291	68864.732	0.992	1987	521				*P* < 0.001	*P* < 0.001
3	69050.529	69341.891	69183.028	0.894	602	1133	773			*P* = 1.000	*P* = 0.998
4	61639.130	62006.247	61806.079	0.841	259	1331	262	656		*P* < 0.001	*P* < 0.001
5	61171.017	61613.887	61372.416	0.831	276	259	625	1086	262	*P* < 0.001	*P* = 0.133

Note: AIC, Akaike information criterion; BIC, Bayesian information criterion; aBIC, adjusted Bayesian information criterion; BLRT, bootstrap likelihood ratio test; aLMR, adjusted Lo–Mendell–Rubin likelihood ratio test

**Fig. 1:**
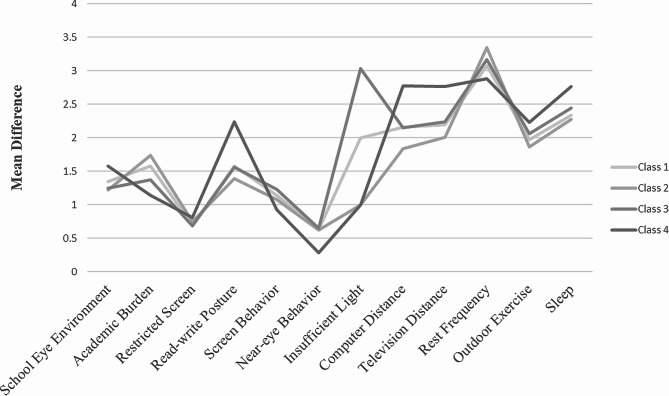
Line chart of latent classes of eye-related behaviors

Figure 1 shows the scores for the latent classes of eye-related behaviors, and Table S1 shows the differences between the latent variables in the four classes. In class 2, academic burden and rest frequency were significantly greater than those in the other classes, and read-write posture, computer distance and television distance were significantly lower than those in the other classes. In class 3, insufficient lighting was significantly greater than in the other classes. In class 4, school eye environment, restricted screen, read-write posture, computer distance, television distance, outdoor activity and sleep time were significantly greater than in the other classes, and academic burden, screen behavior, near-eye behavior and rest frequency were significantly lower than in the other classes. Therefore, four classes of latent eye-related behaviors were obtained and were named the medium health behavior group (class 1, 10.33%), heavy academic burden and near-eye behavior group (class 2, 53.07%), insufficient lighting group (class 3, 10.45%) and high health behavior group (class 4, 26.15%).

### Results of univariate analysis

Table 3 shows the influence of basic demographic characteristics on the distribution of latent eye-related behavior types among junior middle school students. For the sociodemographic characteristics, the distributions of sex (*p* < 0.001), age (*p* < 0.001), survey year (*p* < 0.001) and adolescence (*p* = 0.020) differed among the classes of students. With respect to age, we performed further analysis and found that age was significantly greater in class 1 than in the other classes and was significantly lower in class 4 than in class 1 and class 3 (See Table S2).

**Table 3:** Influence of latent class distribution on eye-related behaviors

Variables	Class 1	Class 2	Class 3	Class 4	χ^2^/ F	*p* value
Gender						
Male	140	672	164	363	14.468	0.002^*^
Female	119	659	98	293		
Age (year)	13.77 ± 0.88	13.59 ± 0.89	13.66 ± 0.89	13.55 ± 0.89	10.712	0.004^*^
Year						
2019	58	358	55	60	148.227	< 0.001^*^
2020	65	345	76	108		
2021	66	308	71	243		
2022	70	320	60	245		
Weight						
Normal	157	781	143	371	7.706	0.314
Overweight	46	291	61	162		
Obesity	56	259	58	123		
Vision						
Normal vision	104	383	107	244	28.870	< 0.001^*^
Myopia	155	948	155	412		
Adolescent						
Yes	103	558	131	306	9.866	0.020^*^
No	156	773	131	350		

Note: *Significant correlation, *P* < 0.05

Table S3 shows the changes in each eye-related behavior between 2019 and 2022 for junior middle school students by sex. Overall, during 2019–2022, the school eye environment (*p* < 0.001), read-write posture (*p* < 0.001), computer distance (*p* = 0.002), and sleep time (*p* < 0.001) significantly increased; academic burden (*p* < 0.001), restricted screen (*p* = 0.001), near-eye behavior (*p* < 0.001), rest frequency (*p* < 0.001), and outdoor exercise (*p* = 0.036) significantly decreased; and differences in screen behavior (*p* = 0.431), insufficient lighting (*p* = 0.084), and television distance (*p* = 0.750) were not significant. Computer distance did not significantly differ among males (*p* = 0.117) and was significantly greater among females (*p* = 0.004). The rest frequency was not significantly different among males (*p* = 0.061) and was significantly lower among females (*p* = 0.041). Outdoor exercise decreased significantly among males (*p* = 0.047) but did not differ significantly among females (*p* = 0.500).

Table S4 shows the distribution of latent eye-related behaviors among junior middle school students by sex. Among the other latent classes, class 2 had the highest number of males, class 1 had the lowest number of males, and class 3 had the lowest number of females. Figure S1 shows the trends in the percentages of the four classes over the period 2019–2022, with class 1 showing an upward and then a downward trend, class 2 showing a downward and then an upward trend, and class 3 and class 4 showing an upward and then a downward trend. Figure S2 shows the change in the proportion of male and female students in the four classes over the period 2019–2022. In class 1, among male students, the proportion increased from 2021 to 2022 compared to 2019–2020, and among female students, the proportion decreased from 2021 to 2022 compared to 2019–2020. Among the respondents in class 2, for each gender, the share in 2021–2022 was lower than that in 2019–2020, while the proportion in 2022 was greater than that in 2021. In class 3, there was an upward and then a downward trend across the four years for each sex, with a peak for males in 2020 and a peak for females in 2021. In class 4, across all genders, there was a significant increase in 2021–2022 compared to 2019–2020, with a peak in 2022.

### Results of the generalized linear model

Table 4 shows the results of the generalized linear model adjusted for age, sex, and adolescence. Students with a heavy academic burden and near-eye behavior (class 2) were 1.466 (95% CI = 1.203 to 1.787, *P* < 0.001) times more likely to be myopic than were those in the healthy group (class 4). After adjusting for age and adolescence, among males, students with heavy academic burdens and near-eye behavior (class 2) were 1.376 (95% CI = 1.056 to 1.793, *P* = 0.018) times more likely to be myopic than were those in the healthy group (class 4); among females, students with heavy academic burdens and eye-related behavior (class 2) were 1.540 (95% CI = 1.141 to 2.078, *P* = 0.005) times more likely to be myopic than were those in the healthy group (class 4).

**Table 4:** Results of generalized linear models of eye-related behaviors

Variables	Class 1 (Refer to class 4)		Class 2 (Refer to class 4)		Class 3 (Refer to class 4)	
	OR (95%CI)	*p* value	OR (95%CI)	*p* value	OR (95%CI)	*p* value
Myopia	0.883 (0.657, 1.185)	0.406	**1.466 (1.203, 1.787)**	**< 0.001**	0.858 (0.640, 1.150)	0.305
Male	0.790 (0.533, 1.170)	0.240	**1.376 (1.056, 1.793)**	**0.018**	0.939 (0.645, 1.366)	0.742
Female	0.973 (0.642, 1.582)	0.973	**1.540 (1.141, 2.078)**	**0.005**	0.772 (0.482, 1.237)	0.282

Notes: CI: confidence interval

## Discussion

Myopia is a common global problem among children and adolescents that requires early prevention and control, and the dual context of the COVID-19 epidemic and the double reduction policy has a significant impact on eye-related behaviour [6], as well as the possibility that students of different genders may have their eye-related behaviours. Previous studies have not generalized the characteristics of latent classes of eye-related behavior among junior middle school students, nor have they found any associations between latent classes of eye-related behavior and myopia in this particular context. In this study, four latent classes of eye-related behaviors among junior middle school students were fitted through LPA, namely, the medium health behavior group, the heavy academic burden and near-eye behavior group, the insufficient lighting group and the high health behavior group. The distribution of the four eye-related behavior subtypes differed among subjects of different genders. Among male students, the highest number (50.19%) were in the heavy academic burden and near-eye behavior group, and the lowest number (10.45%) were in the medium health behavior group; among female students, the highest number (56.37%) were in the heavy academic burden and near-eye behavior group, and the lowest number (8.38%) were in the insufficient lighting group. We found that heavy academic burden and near-eye behavior were risk factors for myopia among junior high school students compared to healthy individuals. These results support our previous hypothesis that different eye-related behaviors will have different effects on the prevention and control of myopia.

During the COVID-19 epidemic, students’ learning and living environments were significantly impacted, producing different changes in eye-related behavior. We found a significant decline in restricting screen behavior between 2019 and 2022, while screen behavior decreased compared to that in 2019, whereas 2020–2022 showed a yearly increase. This is largely driven by students’ home-based online learning, with large-scale web-based online learning becoming the dominant mode of public learning during the COVID-19 pandemic. According to a study in Chongqing, China, 97.7% of primary and middle school students used computers and mobile phones to study online during the COVID-19 pandemic [5]. Screen behavior accelerates the development of myopia and increases the risk of myopia in children and adolescents [15]. In addition, outdoor exercise declined significantly over the four years, especially among males. Xu et al. reported that outdoor activity time decreased by 1.14 h in grades 1–6 and 1.71 h in grades 7–12 during COVID-19 and that outdoor activity time was significantly negatively associated with an increase in the prevalence of myopia during the 6 months before and after COVID-19 [15]. Increased outdoor activity is a protective factor against myopia, helping to slow the growth of the eye axis and thus preventing the development and progression of myopia [16], especially in junior middle school [17]. Therefore, we should focus on students’ screen behavior and outdoor exercise to reduce screen behavior and increase outdoor exercise time, which will be crucial for preventing visual impairment and controlling preexisting visual conditions after COVID-19.

In the Introduction, we asked how the eye-related behavior of children and adolescents in the context of the double reduction policy changed from that before the double reduction policy during the COVID-19 epidemic. In our study, the healthy group exhibited a significant increase after the double reduction, indicating that the double reduction policy had a positive impact on the change in eye-related behavior of junior middle school students, thus effectively promoting their visual health. Compared with those in the predouble reduction period, the heavy academic burden and high near-eye behavior subtypes decreased overall but increased in 2022, mainly due to an increase in near-eye behavior caused by students studying online at home as a result of COVID-19 [18]. Single-eye-related behavior also significantly changed before and after the double reduction, with significant increases in school eye use, read-write posture, computer distance and sleep time and significant decreases in academic burden and near-eye behavior, and these behavioral changes delayed the occurrence and progression of myopia [19–21].However, computer distance did not change significantly among males and increased significantly among females, possibly because males spend more time playing Internet games than females and therefore use computers for long periods at close distances [22]. The rest of the frequency did not change significantly among males and decreased significantly among females, mainly because females are mostly more self-disciplined in their studies than males are and usually spend more time reading and work-related matters [23]. Therefore, we should focus on the sex specificity of different eye-related behaviors in the future and adopt precise interventions for individuals of different genders, e.g., for males, to avoid prolonged close-range use of computers and for females, to increase the rest frequency to avoid prolonged close-range work among junior middle school students.

To our knowledge, this is the first study to explore the relationships between different combinations of eye-related behavior subtypes and myopia among junior high school students of different genders in the context of double reduction during the COVID-19 epidemic. Our fixed surveillance site schools, using four consecutive years of surveillance data covering the pre- and post-COVID-19 epidemic periods, maintained a stable eye-related environment at school, with a representative sample, and the combinations of eye-related behaviors were closer to the reality of junior middle school students, providing an important practical basis and strategic support for myopia prevention and control. Using a latent class model, we demonstrated that different combinations of eye-related behaviors were grouped across genders and that different subtypes of eye-related behavior combinations were grouped heterogeneously for the prevention and control of myopia among junior middle school students. Taken together, these findings suggest that in the prevention and control of myopia among junior middle school students after COVID-19, the government and school should continue to implement a double reduction policy, reduce the academic burden, increase outdoor activities, and achieve early targeted guidance for different genders, providing directions for future interventions as well as priority populations for interventions. In the future, further consideration should be given to the differences in students’ physical fitness when developing individualized and personalized strategies. Although this study has several important findings, it has several limitations. First, based on sentinel surveillance data, this study analyzed the prevalence and progression of myopia in the context of double reduction during the COVID-19 epidemic and the relationship of myopia with different latent classes of eye-related behavior; future intervention studies should be designed to validate and explore the effectiveness of myopia intervention strategies. Second, myopia is caused by a variety of factors, and we have explored only the important environmental factors of unhealthy eye-related behaviors in depth; future detailed analyses should also address genetic factors, substance use and health-related behaviors. Third, we used participants’ self-reported data to analyze this study. Although eye-related behaviors are relatively fixed and recall bias may exist in completing the questionnaire, there is no objective method of assessment. Moreover, the scores of eye-related behaviors and the mean of the scores of the fitted profiles of the model only reflect the relative situation among students, which should be verified in the future in the multi-centre, large-sample dataset.

## Conclusion

The dual reduction policy has a positive effect on improving unhealthy eye-related behaviors, but different eye-related behaviors are gender specific, and the prevention and control of myopia by different subtypes of eye-related behaviors are heterogeneous among junior middle school students. In the post-COVID-19 period, we should continue to implement a double reduction policy, identify the characteristics of different subtypes of eye-related behavior, pay attention to the effects of different subtypes on myopia, and formulate corresponding targeted eye-related behavioral strategies and individualized and personalized programmes to strengthen the prevention and control of myopia, protect the visual health of children and adolescents, and provide an important reference for the prevention and control of myopia among children and adolescents in future public health emergencies.

### Electronic supplementary material

Below is the link to the electronic supplementary material.

Supplementary Material 1

## Data Availability

The datasets generated and/or analysed during the current study are not publicly available due to participants’ privacy but are available from the corresponding author on reasonable request.

## Abbreviations

COVID-19: Coronavirus disease 2019
LPA: Latent profile analysis
LCM: Latent class model
AIC: Akaike information criterion
BIC: Bayesian information criterion
aBIC: Sample-size adjusted Bayesian information criterion
BLRT: Bootstrap likelihood ratio test
aLMR: Adjusted LoMendell–Rubin likelihood ratio test
