# Supplementary material for: Influence of eye-related behavior on myopia among junior middle school students under the background of double reduction during the COVID-19 pandemic
Source: BMC Public Health. 2024 Jun 7;24:1531. doi: 10.1186/s12889-024-18958-0 (PMC11157889; doi:10.1186/s12889-024-18958-0)
Supplement: Supplementary file 1 — Supplementary Material 1 [file 12889_2024_18958_MOESM1_ESM.docx]

**Questionnaire:**

1. School eye environment: How often were class seats changed in the past term? How often is the height of your desk and chair adjusted according to your height, etc.

2. Academic burden: In the past week, how long did you do your homework after school on average? How long did you attend extra-curricular cultural classes, etc.

3. Restriction screen: In the past week, how long did your parents restrict the watching of TV, playing on the computer and other video and screen behaviour?

4. Read-write posture: How often do you pay attention to your reading and writing posture when reading and writing, and how often do you meet the standards? Do your parents or teachers remind you to pay attention to your reading and writing posture?

5. Screen behaviour: In the past week, how long did you watch TV on average each day? How long do you usually use the computer each day?

6. Near-eye behaviour: In the past week, did you look at books or electronic screens in direct sunlight? Do you turn off the lights when you look at electronic screens at night, etc.?

7. Insufficient light: In the past week, how often did you use desk lamps and roof lights at night when reading and writing at home?

8. Computer distance: In the past week, how far were your eyes from the computer monitor when using the computer?

9. Television distance: In the past week, how far were your eyes from the TV monitor when watching TV or playing TV games?

10. Rest frequency: In the past week, how often did you rest your eyes when using your eyes at close range? (e.g. looking away, closing your eyes or playing outdoors, etc.)

11. Outdoor exercise: In the past week, how long did you spend outdoors during the day on average?

12. Sleep: In the past week, how long did you sleep on average each day?


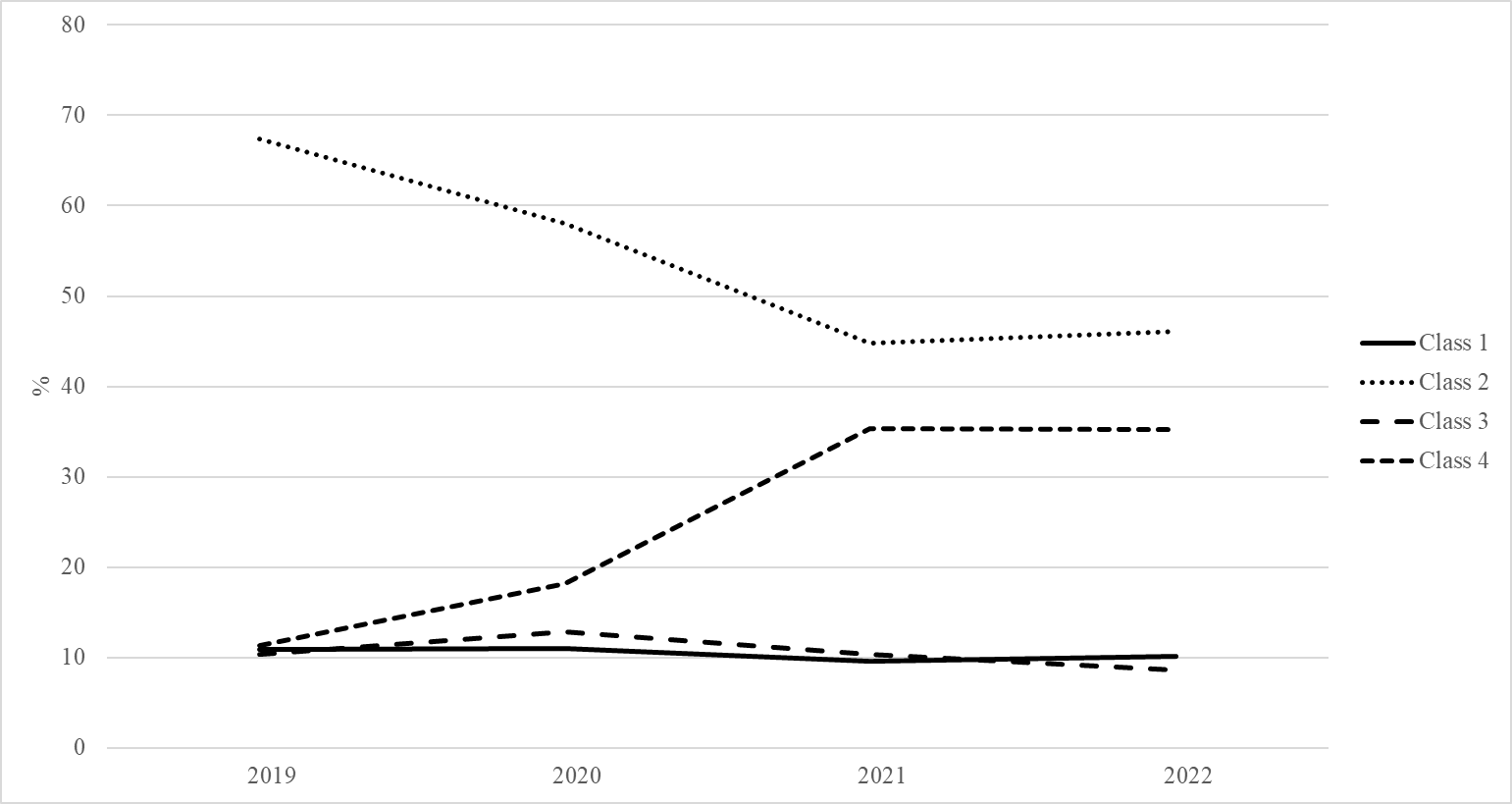
Figure S1 Line chart of latent class distribution during 2019-2022.

Figure S2 Bar chart of latent class distribution in different gender during 2019-2022.


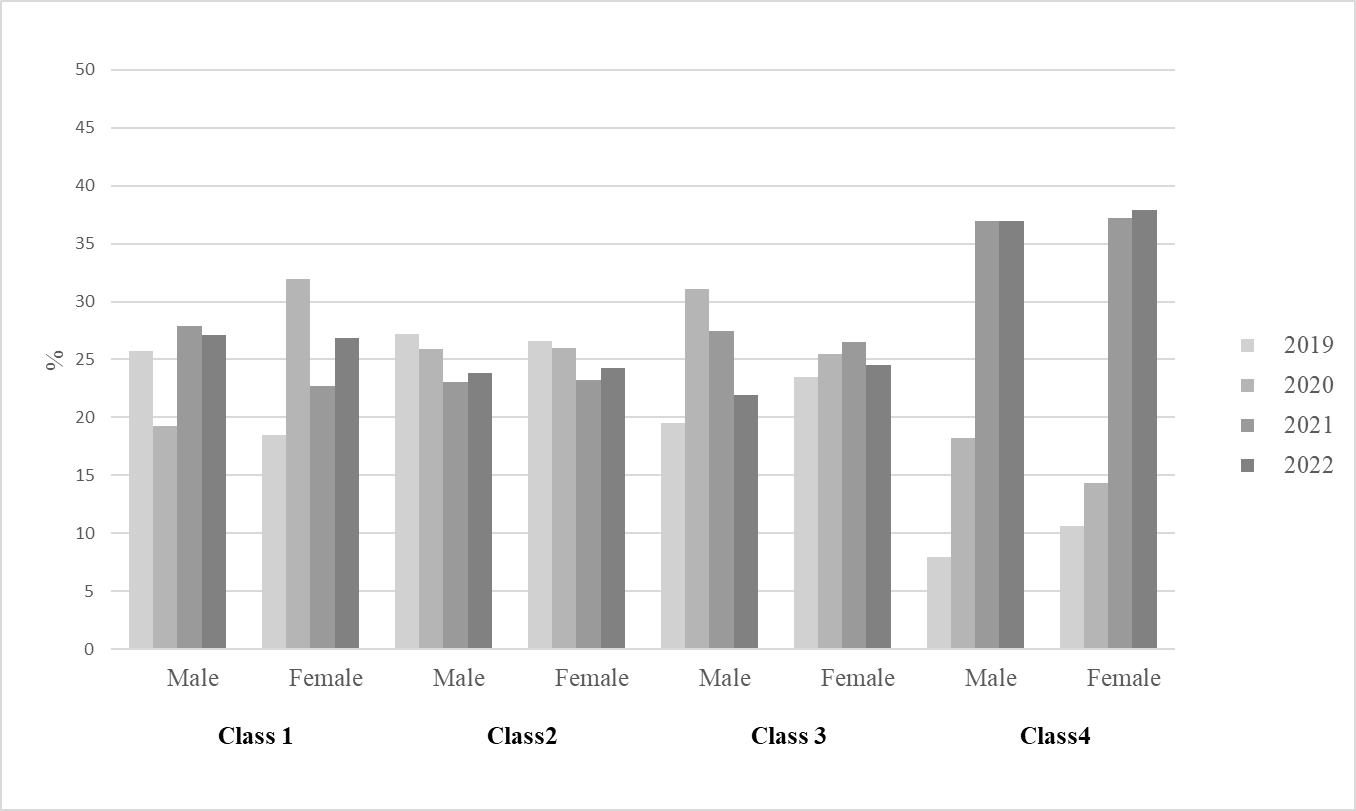


Table S1 Multiple comparisons of age in latent class on eye-related behavior.

|  |  |  | MD | SD | P | 95%CI | |
| --- | --- | --- | --- | --- | --- | --- | --- |
|  |  |  |  |  |  | lower | upper |
| School Eye Environment | Class 1 | Class 2 | 0.12 | 0.03 | <0.01^*^ | 0.06 | 0.19 |
|  |  | Class 3 | 0.09 | 0.01 | 0.03^*^ | 0.01 | 0.18 |
|  |  | Class 4 | -0.26 | 0.04 | <0.01^*^ | -0.33 | -0.19 |
|  | Class 2 | Class 3 | -0.03 | 0.03 | 0.38 | -0.09 | 0.04 |
|  |  | Class 4 | -0.38 | 0.02 | <0.01^*^ | -0.43 | -0.34 |
|  | Class 3 | Class 4 | -0.36 | 0.04 | <0.01^*^ | -0.42 | -0.29 |
| Academic Burden | Class 1 | Class 2 | -0.18 | 0.06 | <0.01^*^ | -0.29 | -0.07 |
|  |  | Class 3 | 0.21 | 0.07 | <0.01^*^ | 0.07 | 0.35 |
|  |  | Class 4 | 0.52 | 0.06 | <0.01^*^ | 0.41 | 0.64 |
|  | Class 2 | Class 3 | 0.39 | 0.06 | <0.01^*^ | 0.28 | 0.50 |
|  |  | Class 4 | 0.70 | 0.04 | <0.01^*^ | 0.63 | 0.78 |
|  | Class 3 | Class 4 | 0.31 | 0.06 | <0.01^*^ | 0.19 | 0.43 |
| Restricted Screen | Class 1 | Class 2 | -0.05 | 0.03 | 0.07 | -0.11 | 0.01 |
|  |  | Class 3 | 0.01 | 0.04 | 0.91 | -0.07 | 0.08 |
|  |  | Class 4 | -0.12 | 0.03 | <0.01^*^ | -0.18 | -0.06 |
|  | Class 2 | Class 3 | 0.06 | 0.03 | 0.04^*^ | 0.01 | 0.11 |
|  |  | Class 4 | -0.07 | 0.02 | <0.01^*^ | -0.11 | -0.03 |
|  | Class 3 | Class 4 | -0.12 | 0.03 | <0.01^*^ | -0.18 | -0.06 |
| Read-write Posture | Class 1 | Class 2 | 0.20 | 0.04 | <0.01^*^ | 0.13 | 0.27 |
|  |  | Class 3 | 0.01 | 0.05 | 0.82 | -0.08 | 0.10 |
|  |  | Class 4 | -0.76 | 0.04 | <0.01^*^ | -0.83 | -0.68 |
|  | Class 2 | Class 3 | -0.19 | 0.04 | <0.01^*^ | -0.26 | -0.12 |
|  |  | Class 4 | -0.96 | 0.03 | <0.01^*^ | -1.00 | -0.91 |
|  | Class 3 | Class 4 | -0.77 | 0.04 | <0.01^*^ | -0.84 | -0.69 |
| Screen Behavior | Class 1 | Class 2 | 0.05 | 0.05 | 0.27 | -0.04 | 0.14 |
|  |  | Class 3 | -0.10 | 0.06 | 0.10 | -0.21 | 0.02 |
|  |  | Class 4 | 0.24 | 0.05 | <0.01^*^ | 0.17 | 0.34 |
|  | Class 2 | Class 3 | -0.15 | 0.04 | <0.01^*^ | -0.23 | -0.06 |
|  |  | Class 4 | 0.19 | 0.03 | <0.01^*^ | 0.13 | 0.25 |
|  | Class 3 | Class 4 | 0.34 | 0.05 | <0.01^*^ | 0.24 | 0.43 |
| Near-eye Behavior | Class 1 | Class 2 | 0.01 | 0.03 | 0.87 | -0.05 | 0.06 |
|  |  | Class 3 | -0.02 | 0.04 | 0.60 | -0.09 | 0.05 |
|  |  | Class 4 | 0.38 | 0.03 | <0.01^*^ | 0.32 | 0.44 |
|  | Class 2 | Class 3 | -0.02 | 0.03 | 0.40 | -0.08 | 0.03 |
|  |  | Class 4 | 0.38 | 0.02 | <0.01^*^ | 0.34 | 0.41 |
|  | Class 3 | Class 4 | 0.40 | 0.03 | <0.01^*^ | 0.34 | 0.46 |
| Insufficient Light | Class 1 | Class 2 | 1.00 | 0.01 | <0.01^*^ | 0.99 | 1.01 |
|  |  | Class 3 | -1.03 | 0.01 | <0.01^*^ | -1.04 | -1.02 |
|  |  | Class 4 | 1.00 | 0.01 | <0.01^*^ | 0.99 | 1.01 |
|  | Class 2 | Class 3 | -2.03 | 0.01 | <0.01^*^ | -2.04 | -2.03 |
|  |  | Class 4 | 0.01 | 0.01 | 1.00 | -0.01 | 0.01 |
|  | Class 3 | Class 4 | 2.03 | 0.01 | <0.01^*^ | 2.03 | 2.04 |
| Computer Distance | Class 1 | Class 2 | 0.34 | 0.09 | <0.01^*^ | 0.16 | 0.51 |
|  |  | Class 3 | 0.01 | 0.12 | 0.93 | -0.22 | 0.24 |
|  |  | Class 4 | -0.70 | 0.10 | <0.01^*^ | -0.89 | -0.51 |
|  | Class 2 | Class 3 | -0.33 | 0.09 | <0.01^*^ | -0.50 | -0.15 |
|  |  | Class 4 | -1.03 | 0.06 | <0.01^*^ | -1.16 | -0.91 |
|  | Class 3 | Class 4 | -0.71 | 0.10 | <0.01^*^ | 0.90 | -0.52 |
| Television Distance | Class 1 | Class 2 | 0.20 | 0.10 | 0.04^*^ | 0.01 | 0.39 |
|  |  | Class 3 | -0.04 | 0.12 | 0.75 | -0.28 | 0.20 |
|  |  | Class 4 | -0.63 | 0.10 | <0.01^*^ | -0.83 | -0.42 |
|  | Class 2 | Class 3 | -0.24 | 0.10 | 0.01^*^ | -0.43 | -0.05 |
|  |  | Class 4 | -0.82 | 0.07 | <0.01^*^ | -0.96 | -0.69 |
|  | Class 3 | Class 4 | -0.59 | 0.10 | <0.01^*^ | -0.79 | -0.38 |
| Rest Frequency | Class 1 | Class 2 | -0.28 | 0.09 | <0.01^*^ | -0.45 | -0.11 |
|  |  | Class 3 | -0.10 | 0.11 | 0.40 | -0.31 | 0.12 |
|  |  | Class 4 | 0.24 | 0.09 | 0.01^*^ | 0.06 | 0.42 |
|  | Class 2 | Class 3 | 0.18 | 0.09 | 0.03^*^ | 0.02 | 0.35 |
|  |  | Class 4 | 0.52 | 0.06 | <0.01^*^ | 0.40 | 0.64 |
|  | Class 3 | Class 4 | 0.34 | 0.09 | <0.01^*^ | 0.15 | 0.52 |
| Outdoor Exercise | Class 1 | Class 2 | 0.11 | 0.07 | 0.10 | -0.02 | 0.25 |
|  |  | Class 3 | -0.09 | 0.09 | 0.33 | -0.26 | 0.09 |
|  |  | Class 4 | -0.30 | 0.08 | <0.01^*^ | -0.45 | -0.15 |
|  | Class 2 | Class 3 | -0.20 | 0.07 | <0.01^*^ | -0.34 | -0.07 |
|  |  | Class 4 | -0.41 | 0.05 | <0.01^*^ | -0.51 | -0.32 |
|  | Class 3 | Class 4 | -0.21 | 0.08 | <0.01^*^ | -0.36 | -0.06 |
| Sleep | Class 1 | Class 2 | 0.07 | 0.06 | 0.24 | -0.04 | 0.18 |
|  |  | Class 3 | -0.10 | 0.07 | 0.16 | -0.25 | 0.04 |
|  |  | Class 4 | -0.46 | 0.06 | <0.01^*^ | -0.58 | -0.34 |
|  | Class 2 | Class 3 | -0.17 | 0.06 | <0.01^*^ | -0.28 | -0.06 |
|  |  | Class 4 | -0.53 | 0.04 | <0.01^*^ | -0.61 | -0.45 |
|  | Class 3 | Class 4 | -0.36 | 0.06 | <0.01^*^ | -0.48 | -0.24 |

Notes: MD, mean differences; SD, standard deviation; CI, confidence interval.

Table S2 Multiple comparisons of age in latent class on eye-related behavior.

|  |  |  | MD | SD | P | 95%CI | |
| --- | --- | --- | --- | --- | --- | --- | --- |
|  |  |  |  |  |  | lower | upper |
| Age | Class 1 | Class 2 | 0.18 | 0.06 | <0.01^*^ | 0.06 | 0.30 |
|  |  | Class 3 | 0.11 | 0.08 | 0.17 | -0.04 | 0.26 |
|  |  | Class 4 | 0.23 | 0.06 | <0.01^*^ | 0.10 | 0.35 |
|  | Class 2 | Class 3 | -0.08 | 0.06 | 0.21 | -0.19 | 0.04 |
|  |  | Class 4 | 0.04 | 0.04 | 0.32 | -0.04 | 0.13 |
|  | Class 3 | Class 4 | 0.12 | 0.07 | 0.07 | -0.01 | 0.25 |

Notes: MD, mean differences; SD, standard deviation; CI, confidence interval.

*Significant correlation, P<0.05.

Table S3 Eye-related behaviors of different genders among junior middle school students during 2019-2022.

| Variables | 2019 | 2020 | 2021 | 2022 | *F* | *p* value |
| --- | --- | --- | --- | --- | --- | --- |
| School Eye Environment | 1.19 ± 0.51 | 1.24 ± 0.49 | 1.42 ± 0.48 | 1.44 ± 0.50 | 38.041 | <0.001^*^ |
| Male | 1.21 ± 0.53 | 1.30 ± 0.51 | 1.46 ± 0.49 | 1.45 ± 0.53 | 17.915 | <0.001^*^ |
| Female | 1.18 ± 0.49 | 1.18 ± 0.46 | 1.38 ± 0.47 | 1.43 ± 0.47 | 22.140 | <0.001^*^ |
| Academic Burden | 2.07 ± 0.84 | 1.94 ± 0.80 | 1.05 ± 0.68 | 1.19 ± 0.68 | 294.285 | <0.001^*^ |
| Male | 2.00 ± 0.84 | 1.86 ± 0.81 | 1.03 ± 0.68 | 1.17 ± 0.68 | 138.711 | <0.001^*^ |
| Female | 2.15 ± 0.83 | 2.04 ± 0.78 | 1.08 ± 0.68 | 1.22 ± 0.68 | 158.361 | <0.001^*^ |
| Restricted Screen | 0.79 ± 0.41 | 0.77 ± 0.42 | 0.77 ± 0.42 | 0.70 ± 0.46 | 5.399 | 0.001^*^ |
| Male | 0.80 ± 0.40 | 0.79 ± 0.41 | 0.78 ± 0.42 | 0.72 ± 0.45 | 2.733 | 0.042^*^ |
| Female | 0.77 ± 0.42 | 0.74 ± 0.44 | 0.76 ± 0.43 | 0.68 ± 0.47 | 2.814 | 0.038^*^ |
| Read-write Posture | 1.53 ± 0.61 | 1.63 ± 0.63 | 1.74 ± 0.69 | 1.72 ± 0.70 | 12.069 | <0.001^*^ |
| Male | 1.58 ± 0.62 | 1.68 ± 0.64 | 1.73 ± 0.67 | 1.74 ± 0.72 | 3.809 | 0.010^*^ |
| Female | 1.48 ± 0.60 | 1.58 ± 0.62 | 1.75 ± 0.70 | 1.70 ± 0.68 | 9.462 | <0.001^*^ |
| Screen Behavior | 1.10 ± 0.69 | 1.04 ± 0.66 | 1.05 ± 0.67 | 1.06 ± 0.67 | 0.919 | 0.431 |
| Male | 1.13 ± 0.75 | 1.07 ± 0.68 | 1.09 ± 0.67 | 1.06 ± 0.67 | 0.685 | 0.561 |
| Female | 1.06 ± 0.61 | 1.01 ± 0.64 | 0.99 ± 0.66 | 1.07 ± 0.68 | 1.074 | 0.359 |
| Near-eye Behavior | 0.63 ± 0.48 | 0.55 ± 0.46 | 0.50 ± 0.43 | 0.47 ± 0.45 | 14.789 | <0.001^*^ |
| Male | 0.60 ± 0.48 | 0.54 ± 0.44 | 0.53 ± 0.42 | 0.46 ± 0.45 | 4.879 | 0.002^*^ |
| Female | 0.67 ± 0.48 | 0.56 ± 0.47 | 0.48 ± 0.43 | 0.47 ± 0.45 | 11.747 | <0.001^*^ |
| Insufficient Light | 1.33 ± 0.68 | 1.37 ± 0.71 | 1.30 ± 0.65 | 1.28 ± 0.62 | 2.222 | 0.084 |
| Male | 1.36 ± 0.70 | 1.41 ± 0.77 | 1.35 ± 0.69 | 1.30 ± 0.65 | 1.455 | 0.225 |
| Female | 1.28 ± 0.66 | 1.32 ± 0.63 | 1.25 ± 0.60 | 1.25 ± 0.59 | 0.849 | 0.467 |
| Computer Distance | 2.05 ± 1.36 | 2.14 ± 1.41 | 2.12 ± 1.41 | 2.33 ± 1.35 | 4.904 | 0.002^*^ |
| Male | 2.08 ± 1.36 | 2.27 ± 1.40 | 2.23 ± 1.38 | 2.33 ± 1.32 | 1.968 | 0.117 |
| Female | 2.02 ± 1.36 | 1.98 ± 1.41 | 1.98 ± 1.43 | 2.33 ± 1.38 | 4.525 | 0.004^*^ |
| Television Distance | 2.22 ± 1.39 | 2.29 ± 1.46 | 2.23 ± 1.47 | 2.29 ± 1.46 | 0.404 | 0.750 |
| Male | 2.14 ± 2.35 | 2.39 ± 1.44 | 2.30 ± 1.43 | 2.33 ± 1.47 | 1.699 | 0.165 |
| Female | 2.31 ± 1.44 | 2.18 ± 1.49 | 2.15 ± 1.51 | 2.24 ± 1.46 | 0.643 | 0.587 |
| Rest Frequency | 3.27 ± 1.36 | 3.29 ± 1.35 | 3.11 ± 1.24 | 3.05 ± 1.20 | 5.176 | 0.001^*^ |
| Male | 3.23 ± 1.38 | 3.27 ± 1.28 | 3.09 ± 1.19 | 3.05 ± 1.18 | 2.467 | 0.061 |
| Female | 3.31 ± 1.35 | 3.32 ± 1.42 | 3.14 ± 1.30 | 3.06 ± 1.23 | 2.754 | 0.041^*^ |
| Outdoor Exercise | 1.92 ± 1.06 | 2.07 ± 1.07 | 2.05 ± 1.02 | 1.96 ± 1.01 | 2.845 | 0.036^*^ |
| Male | 1.91 ± 1.07 | 2.09 ± 1.03 | 2.04 ± 1.02 | 1.91 ± 1.01 | 2.656 | 0.047^*^ |
| Female | 1.93 ± 1.06 | 2.04 ± 1.11 | 2.05 ± 1.04 | 2.01 ± 1.00 | 0.789 | 0.5 |
| Sleep | 2.35 ± 0.84 | 2.22 ± 0.80 | 2.64 ± 0.87 | 2.49 ± 0.90 | 28.244 | <0.001^*^ |
| Male | 2.36 ± 0.83 | 2.27 ± 0.78 | 2.68 ± 0.84 | 2.57 ± 0.87 | 17.406 | <0.001^*^ |
| Female | 2.34 ± 0.85 | 2.16 ± 0.81 | 2.58 ± 0.90 | 2.40 ± 0.93 | 11.748 | 0.001^*^ |

Table S4 Distribution of latent categories and gender of junior middle school students with different eye-related behaviours.

| Variables | Class 1 | Class 2 | Class 3 | Class 4 |
| --- | --- | --- | --- | --- |
| Male | 140 (10.45%) | 672 (50.19%) | 164 (12.25%) | 363 (27.11%) |
| Female | 119 (10.18%) | 659 (56.37%) | 98 (8.38%) | 293 (25.07%) |
